# Morphological, molecular, and pharmacological review of veldt grape (*Cissus quadrangularis* L.): an underutilized medicinal plant

**DOI:** 10.3389/fpls.2025.1586624

**Published:** 2025-06-09

**Authors:** K. Vinoth, S. Ramesh Kumar

**Affiliations:** Department of Horticulture and Food Science, VIT School of Agricultural Innovations and Advanced Learning, Vellore Institute of Technology, Vellore, Tamil Nadu, India

**Keywords:** veldt grape, osteoblast activity, CAM pathway, morphological, molecular

## Abstract

*Cissus quadrangularis*, commonly known as veldt grape, is an underutilized medicinal plant belonging to the Vitaceae family, widely found in tropical regions of India with diverse populations. The plant is also known for its rich phytochemical profile, including ketosteroids and flavonoids that enhance osteoblast activity and accelerate fracture healing. Also, this genus is unique in having a high degree of drought tolerance due to its facultative crassulacean acid metabolism (CAM) pathway. The CAM pathway in *C. quadrangularis* enhances its ability to sustain metabolic activity under drought stress, contributing to its survival and medicinal value in resource-limited ecosystems. The genetic variability among various veldt grape ecotypes plays a crucial role in determining the concentration of bioactive compounds and other physiological traits. This review aims to understand the relationship through morphological and molecular aspects of this plant and its application in pharmacology, with other potential therapeutic applications. *C. quadrangularis*, being an under-utilized medicinal crop with enormous pharmaceutical significance, lacks knowledge of its genetic wealth poses a major barrier to commercial cultivation all over India. Hence, the morphological and molecular markers are a perfect approach to conserving this valuable plant’s genetic resources and crop improvement.

## Introduction

Veldt grape is a perennial climber and succulent plant widely spread throughout Africa and Southeast Asia. It is famously known for its wide adaptability under severe dry conditions due to the facultative crassulacean acid metabolism pathway (Ting et al., 1983). This kind of plant was of great concern and the reason that scientific circles have extensively employed it in human and veterinary medical applications. Dry stems constitute high-quality material for export to various pharmaceutical industries. It can be useful in burning fat, enlarging lean muscle mass, and reducing appetite. It also reduces cholesterol levels, possibly because of its notable effect on metabolic functions in the body. It can shield the cardiovascular system from stress, atherosclerosis, myocardial infarction, strokes, and hypertension by decreasing the bad cholesterol in the human body (Oben et al., 2006). Veldt grape extract provides a high source of calcium ions, which interact with carbon dioxide that leads to calcite crystal formation, indicating that bioorganic molecules of the extract modulate the crystal morphology (Sanyal et al., 2005). According to the Indian Council of Medical Research (ICMR), the daily requirement of calcium is about 600 mg for both sexes of the human population. The daily average intake of calcium is about 900–1000 mg in the USA and Northern Europe, while in India and South Asia, it is only about 400–500 mg. Owing to its profuse medicinal uses, veldt grape has been habitually used by several ethnic groups in India, mainly for its crack recuperating potential for broken bones. Apart from this, the plant is used to treat diabetes and obesity with the multitude of medicinal compounds in its vegetative sections (Jainu and Devi, 2005). Improvement of quantitative traits completely depends on the magnitude of genetic variability and the heritability of desirable characters in the formulation of a plant breeding program. Hereditary inconsistency and ecotype assortment are crucial for the selection of a superior performer among the genotypes (Adugna and Labuschagne, 2003). **Table 1** depicts the scientific classification of *Cissus quadrangularis* (Shukla et al., 2015).

**Table 1:** Scientific classification of *C. quadrangularis* (Shukla et al., 2015).

Kingdom	Plantae
Subkingdom	Tracheobionta
Super division	spermatophyta
Division	Magnoliophyta
Class	Magnoliopsida
Subclass	Rosidae
Order	Vitales
Family	Vitaceae
Genus	*Cissus*
Species	*quadrangularis*

**Figure 1:**
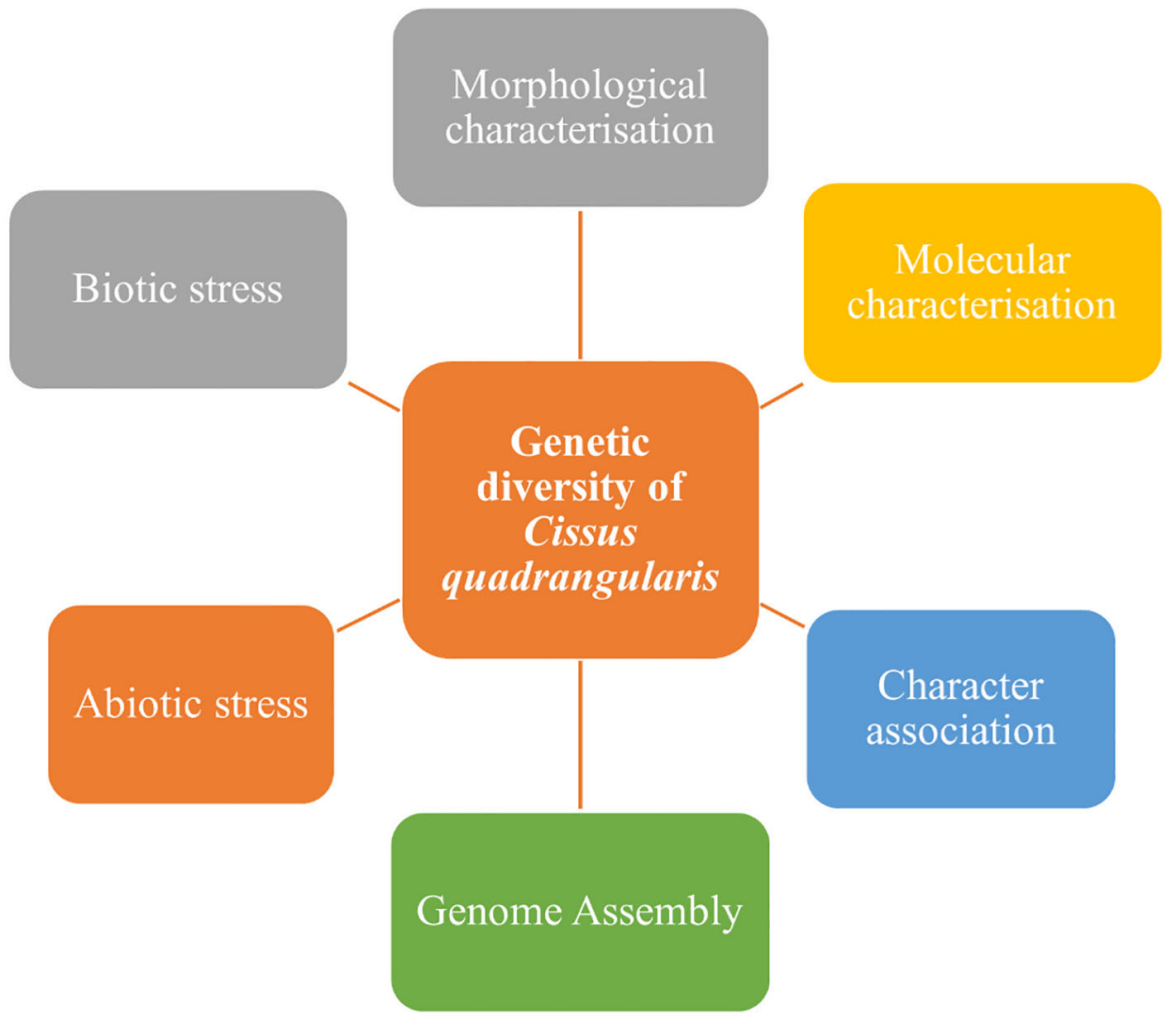
Genetic diversity of *C. quadrangularis*.

The current review is carried out to understand the existence of genetic variability through morphological and molecular characterization by using DNA molecular markers with their potential uses of biochemicals for the further selection program. **Figure 1** explains the outline of the genetic diversity of *C. quadrangularis.*

## Wild relatives and cultivated types/species

*Cissus* is the genus of the Vitaceae family, consisting of 800 species divided into 13 genera throughout the world, including Africa, Arabia, South Asia, Sri Lanka, India, and other tropical regions. Out of these, 8 genera and 63 different species are found in India (Ansarali et al., 2016. **Table 2** depicts the chromosome numbers, ploidy types, and genome size in various species

**Table 2:** Wild relatives and cultivated types/species (Chu et al., 2018a; Raghavan, 1957; Gichuki DK).

*Cissus* species	Chromosome numbers (2*n*)	Ploidy type	Genome size (2C-value)
*C. rotundifolia Vahl*	24	2 ×	0.76 pg
*C. discolor Blume*	24	2 ×	0.86 pg
*C. tuberosa Moc. & Sesse ex DC.*	24	2 ×	0.9 pg
*C. javana DC.*	–	–	0.74 pg
*C. antarctica Vent*	40	4 ×	1.34 pg
*C. trifoliata (L.) L*	48	4 ×	1.58 pg
*C. microcarpa Vahl*	66	6 ×	2.06 pg
*C. quadrangularis* - Raghavan (1957)	24	2 ×	–
*C. quadrangularis* -Robert et al. (2001)	24, 28	–	–
*C. quadrangularis* - Gichuki et al. (2019a)	48	–	1.410 pg

## Morphological characteristics of *C. quadrangularis*

According to Kavitha et al. (2007), morphological characteristics offer participants with unique identification of genotypes that can be utilized as preliminary screening descriptors. Genomic interactions of cultivars with certain environmental factors are adequately captured through these elements, as indicated by Patterson and Weatherup (1984). As per Shah (2011), morphological traits such as leaf, stem, tendril, and root showcased significant levels of disparity among the major variants of the *C. quadrangularis*.

### Habitat

*C. quadrangularis* is widely distributed all over hotter parts of Tamil Nadu, growing in slopy elevations up to 500 MSL with an annual rainfall of less than 700 mm. Maximum diversity is observed in Virudhunagar, Tirunelveli, Tuticorin, Perambalur, Salem, and Erode districts of Tamil Nadu (Rajendran and Agarwal, 2007). It is a rambling shrub or vine, characterized by a thick, four-sided, fleshy stem. Phyllotaxy is alternate and distichous (Jackes and , 1988b).

### Leaves

**Figure 2:**
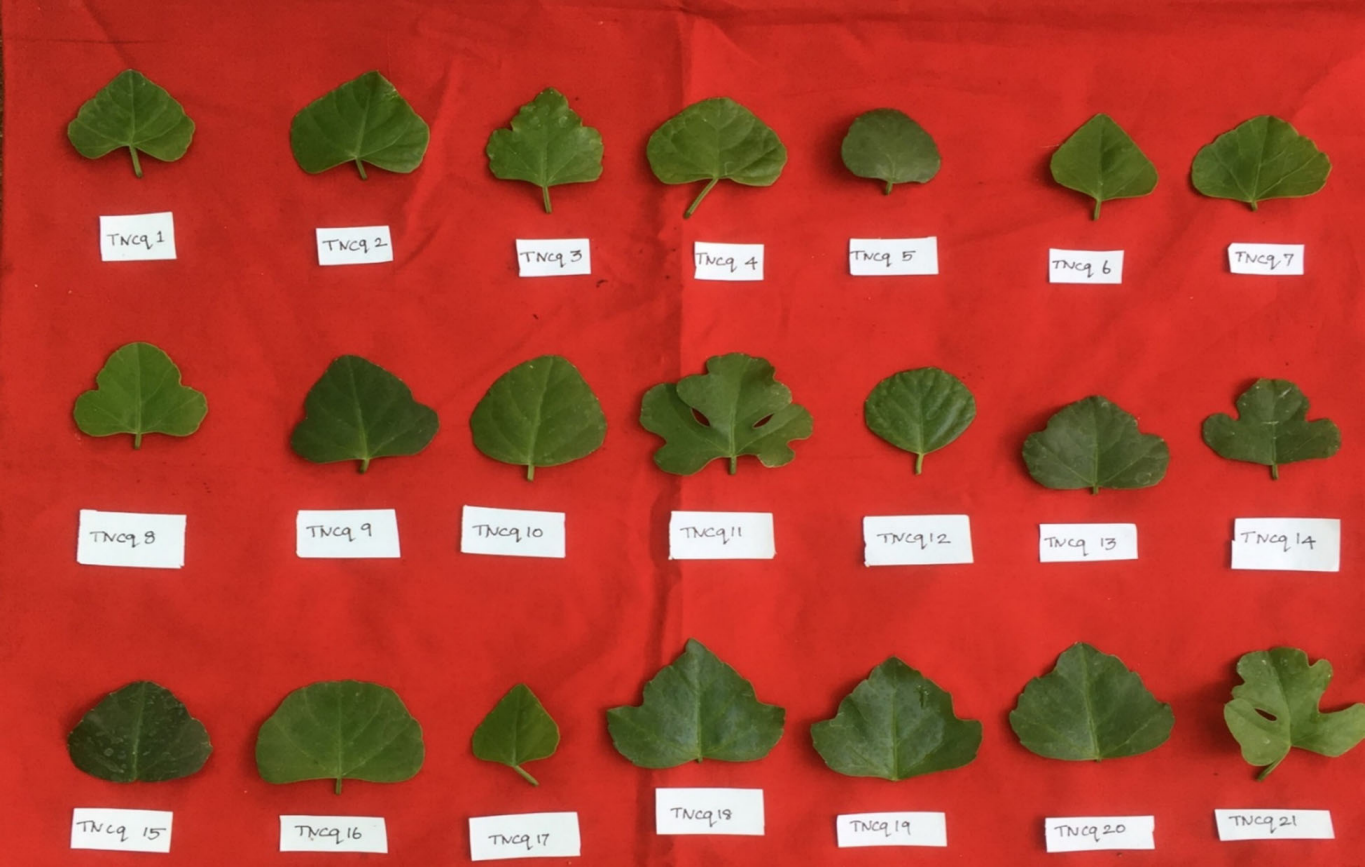
Morphological variability of leaves observed from various districts of Tamil Nadu, India. TNCq, Tamil Nadu *Cissus quadrangularis* 1 (Vinoth et al., 2021).

**Figure 3:**
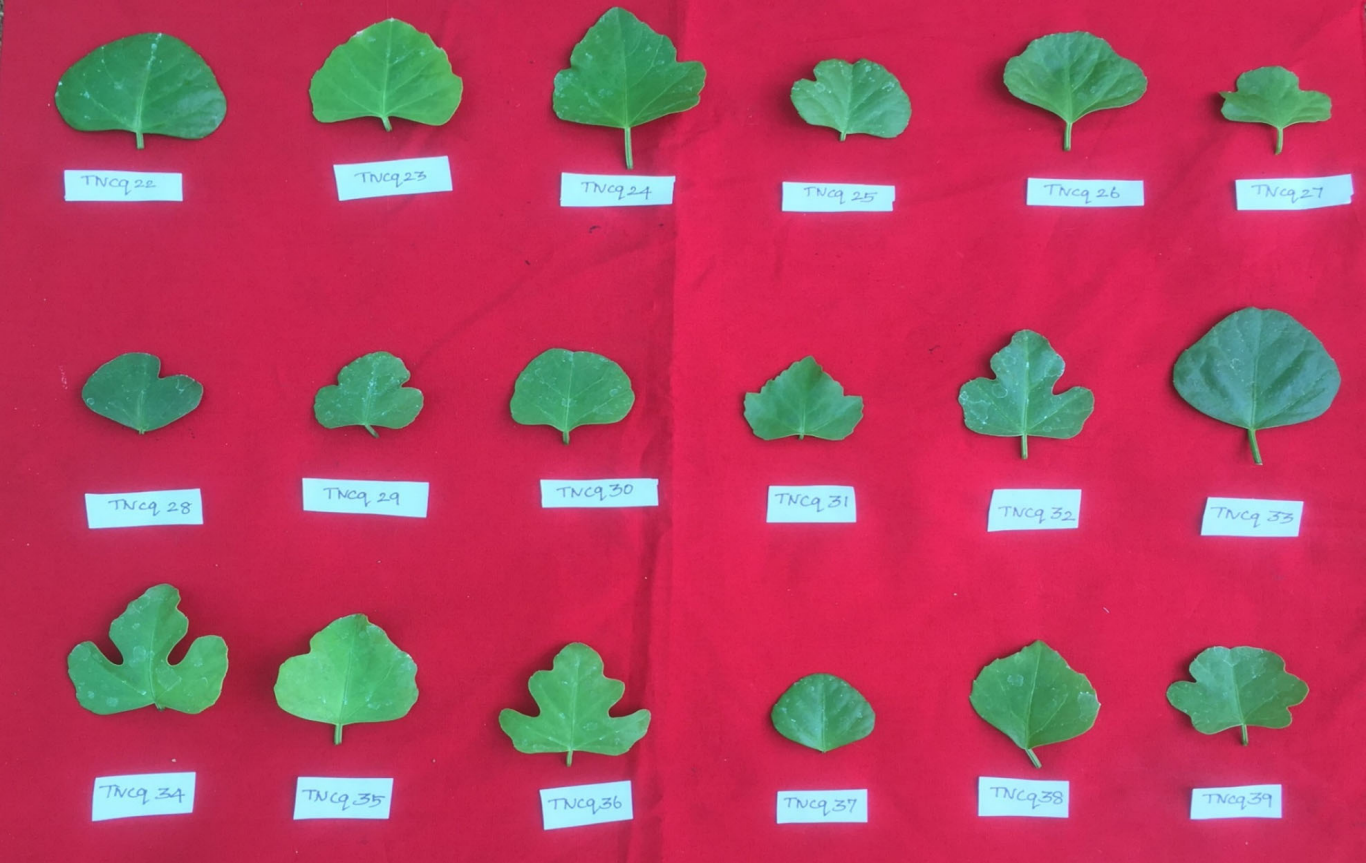
Morphological variability of leaves observed from various districts of Tamil Nadu, India. TNCq, Tamil Nadu *Cissus quadrangularis* 1 (Vinoth et al., 2021).

**Figure 4:**
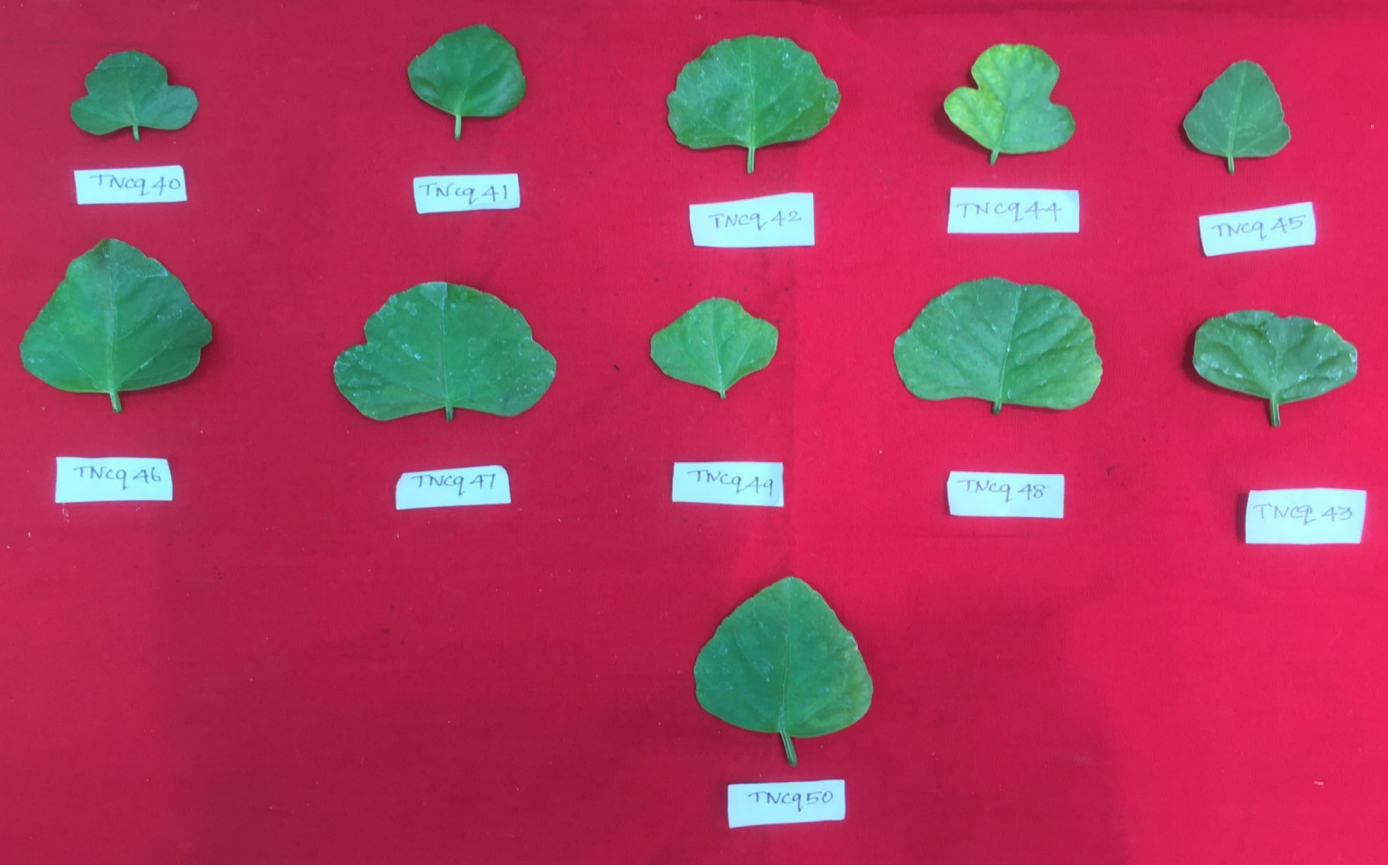
Morphological variability of leaves observed from various districts of Tamil Nadu, India. TNCq, Tamil Nadu *Cissus quadrangularis* 1 (Vinoth et al., 2021).

Leaves are simple, ovate, or reniform, with a ±5-cm-wide base and truncate cordate. Petiole is ±2 cm long (Panda et al, 2004). It is also found that there are numerous morphological differences in petioles with moderate green color and more kidney-shaped leaves to pentagonal-shaped leaves with greenish pink-colored flowers. **Figures 2**–**4** show the morphological variability observed in leaves from various districts of Tamil Nadu, India.

### Stem

**Figure 5:**
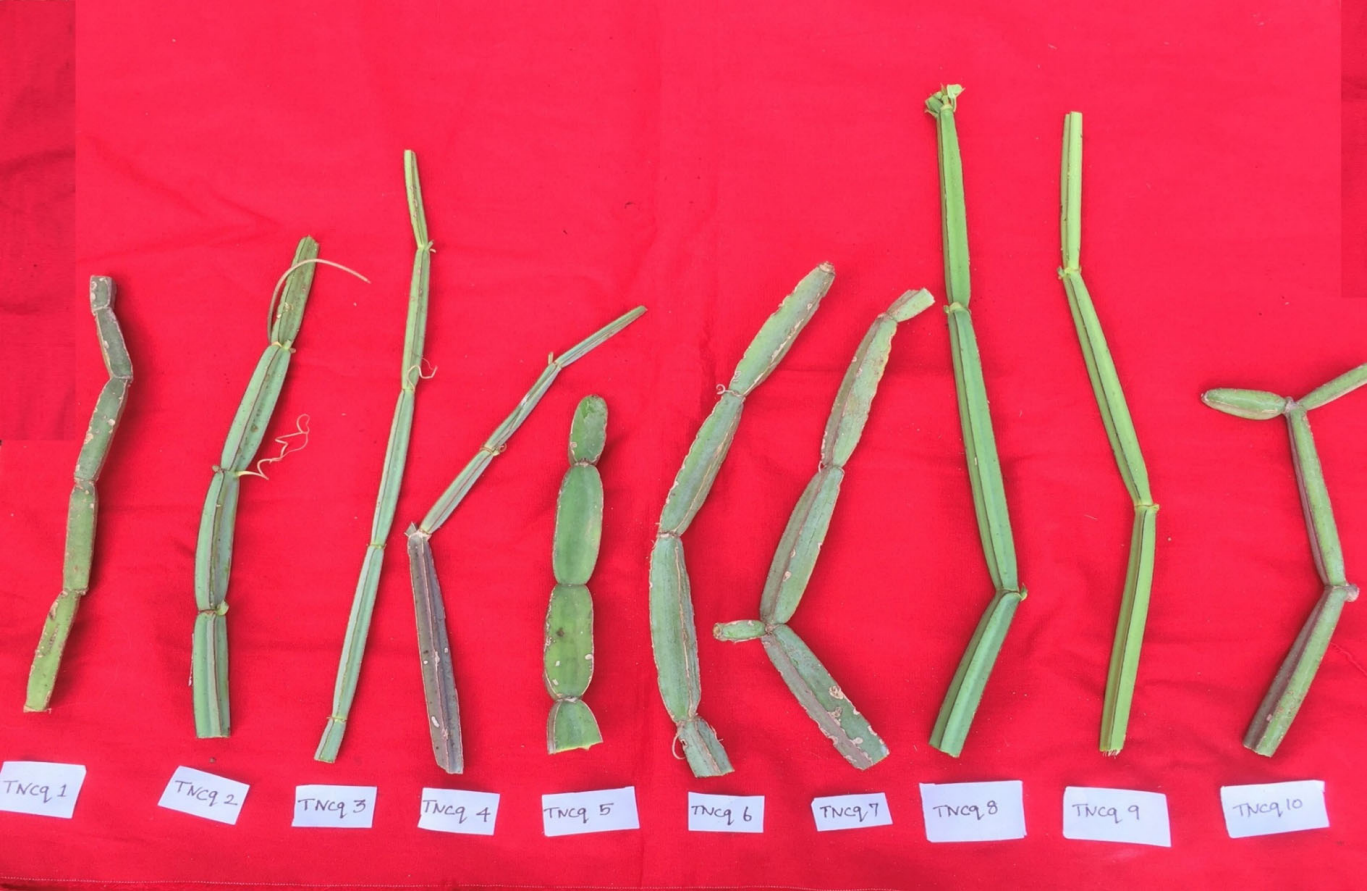
Morphological variability of stems observed from various districts of Tamil Nadu, India. *TNCq, Tamil Nadu *Cissus quadrangularis* 1 (Vinoth et al., 2021).

**Figure 6:**
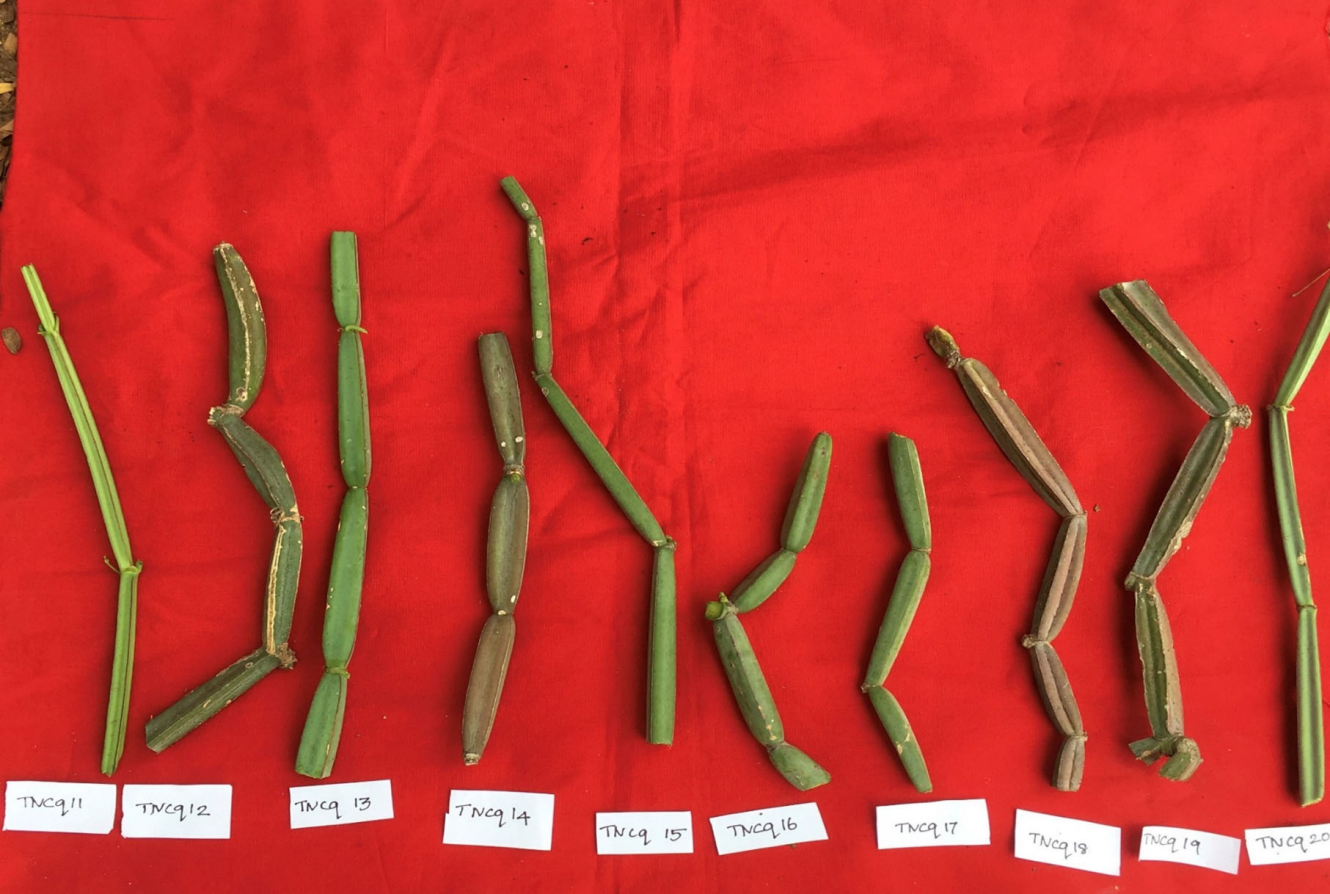
Morphological variability of stems observed from various districts of Tamil Nadu, India. TNCq, Tamil Nadu *Cissus quadrangularis* 1 (Vinoth et al., 2021).

**Figure 7:**
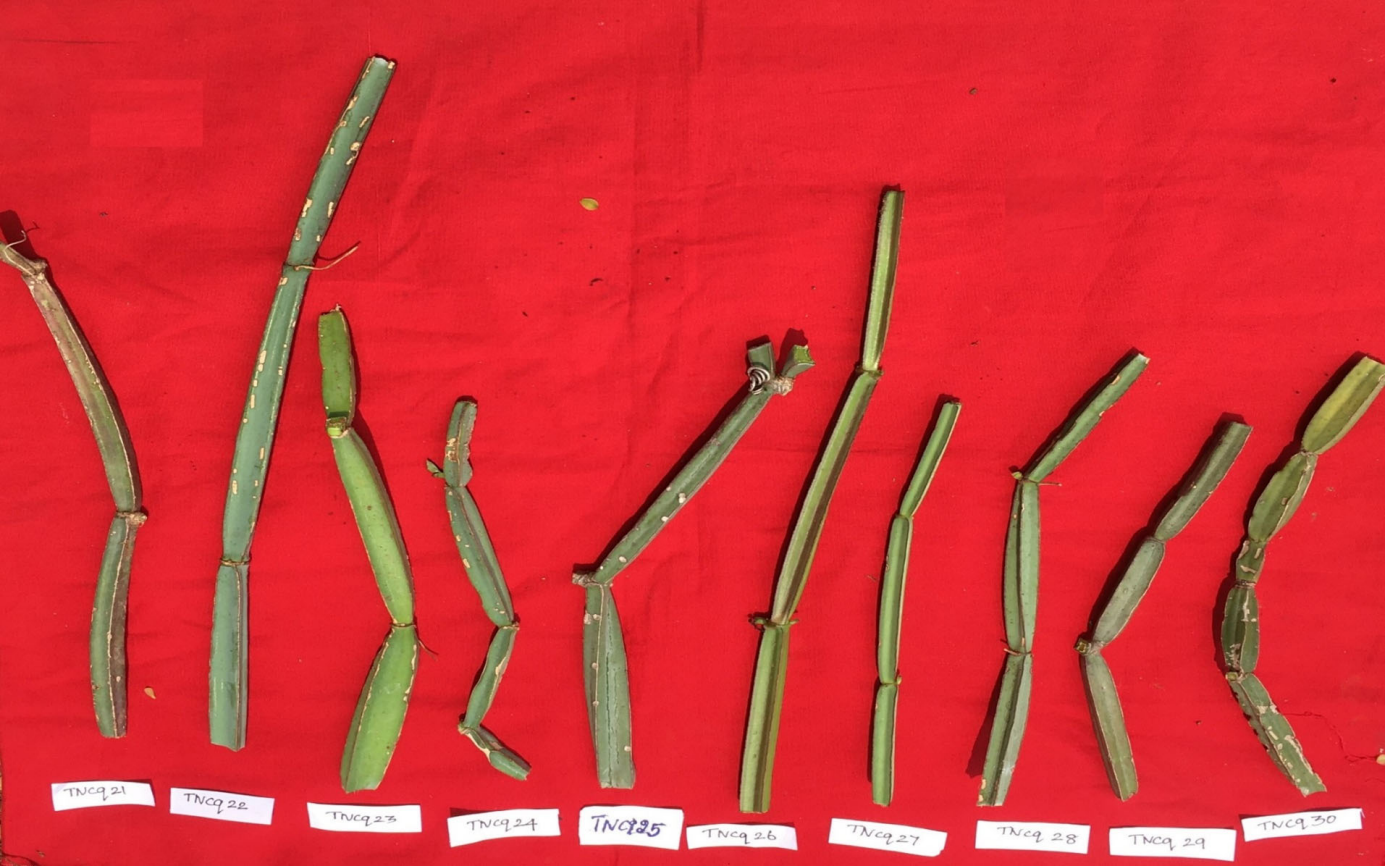
Morphological variability of stems observed from various districts of Tamil Nadu, India. TNCq, Tamil Nadu *Cissus quadrangularis* 1 (Vinoth et al., 2021).

**Figure 8:**
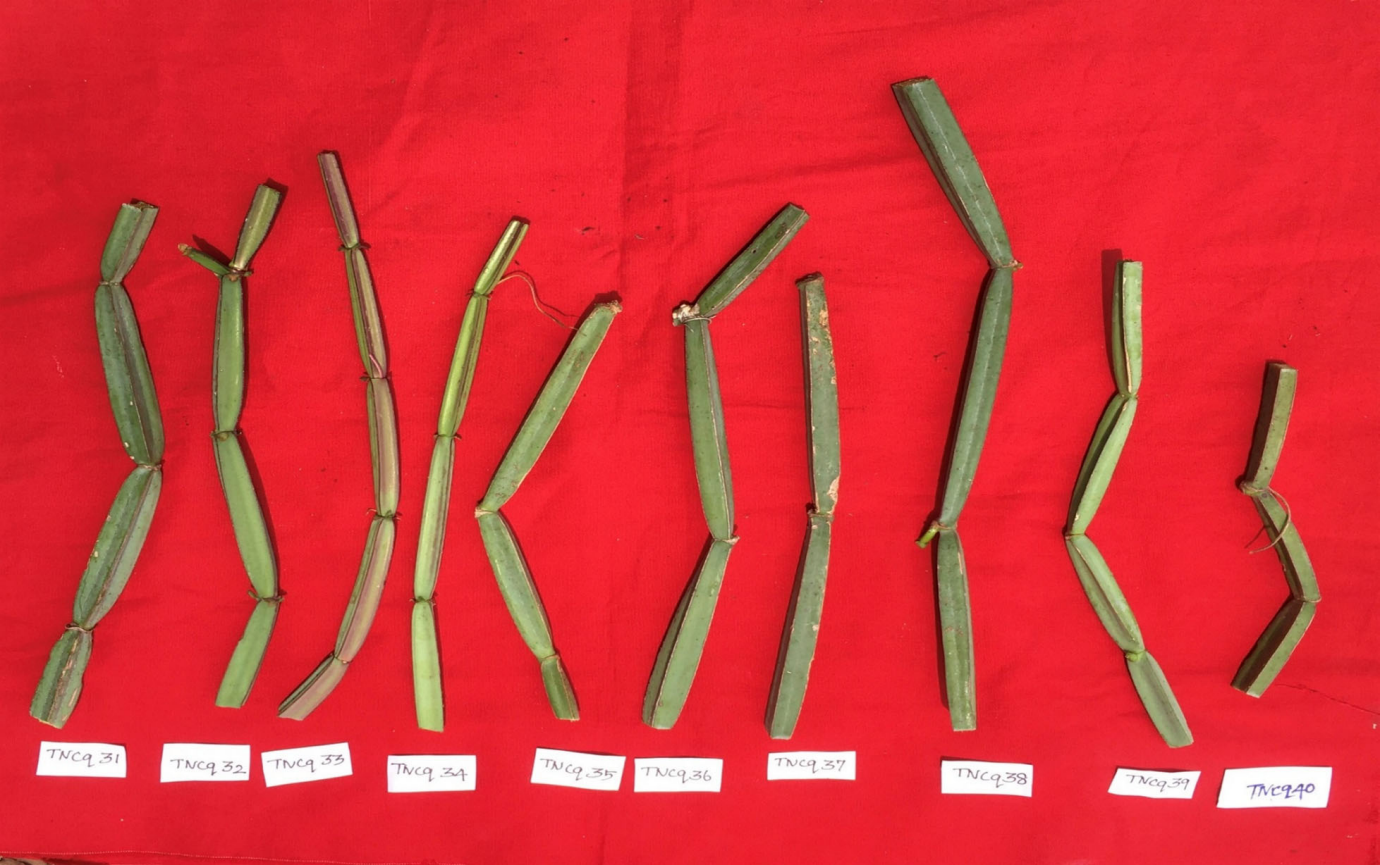
Morphological variability of stems observed from various districts of Tamil Nadu, India. TNCq, Tamil Nadu *Cissus quadrangularis* 1 (Vinoth et al., 2021).

Plant material appears in fragments of various sizes; stems are four-angled and four-winged, and internodes range between 4 and 15 cm in length and between 1 and 2 cm thick. Its surface is devoid of pubescence, glabrous, and has a buff color with a slight greenish blue, while the angular part has a reddish-brown color; it is tasteless and odorless. **Figures 5**–**8** depict morphological differences in stems (dark green to light green) (Vinoth et al., 2021). Three morphovariants of *C. quadrangularis* with square-stemmed, round-stemmed, and flat-stemmed plants are available, which are differentiated as variants I, II, and III, respectively (Austin et al., 2004). Interestingly, three morphotypes (morphotype I, morphotype II, and morphotype III) have been identified based on stem morphology. Morphovariant I and morphovariant III constituted the same, whereas morphovariant II, having a flattened stem, was different (Kaur and Malik, 2014).

### Root, flowers, seed, and berry

The tap-root is 10–20 cm. Flowering of *C. quadrangularis* is from June to December. Flowers are in short peduncle cymes with spreading umbellate branches. The calyx is cup-shaped, truncate, or very obscurely lobed. Petals are four, ovate-oblong, short, and stout. The seed is small with a thick testa (Mahadik, 2021).

**Figure 9:**
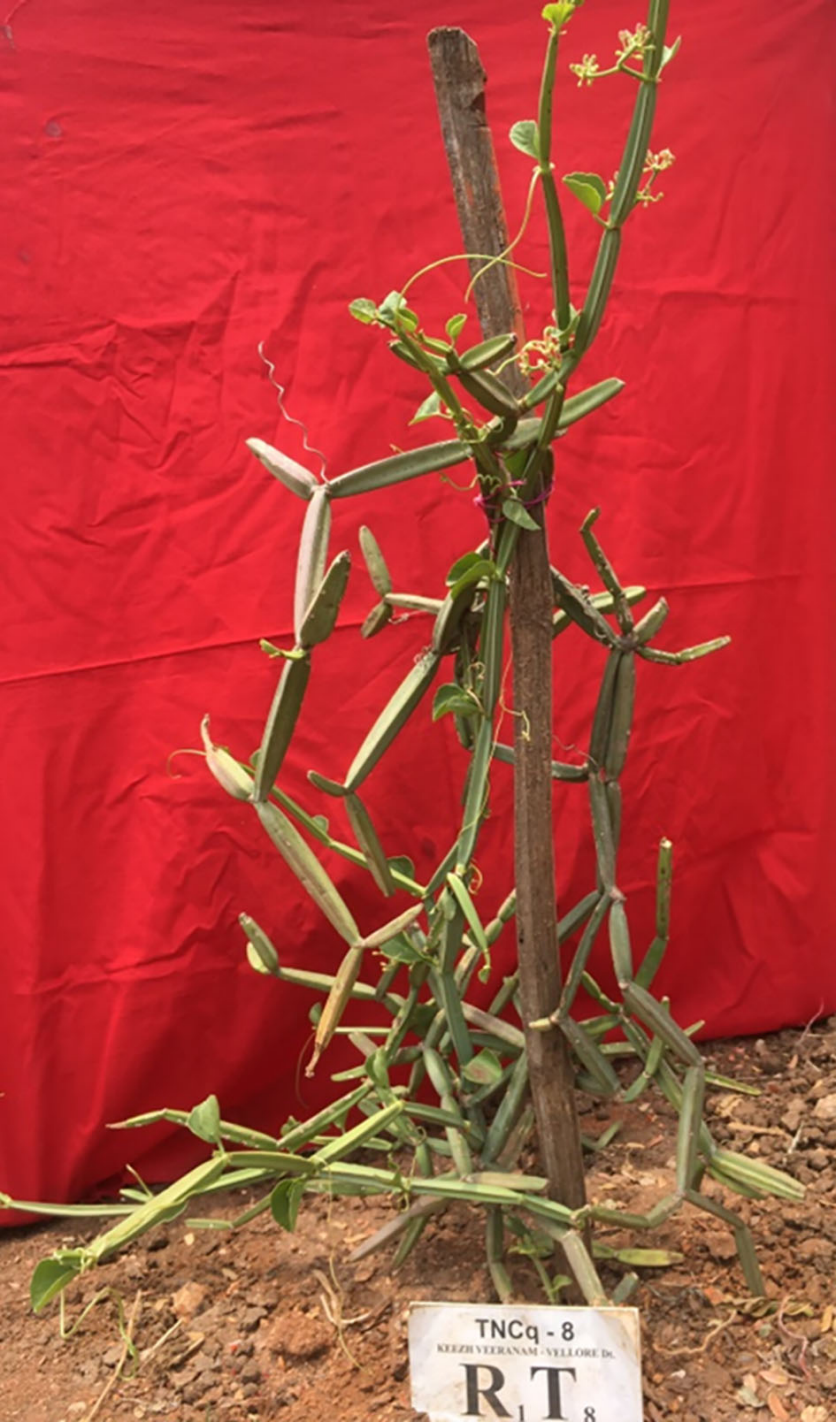
TNCq8 — Flowering ecotypes *C. quadrangularis*. TNCq, Tamil Nadu *Cissus quadrangularis* 1 (Vinoth et al., 2021).

**Figure 10:**
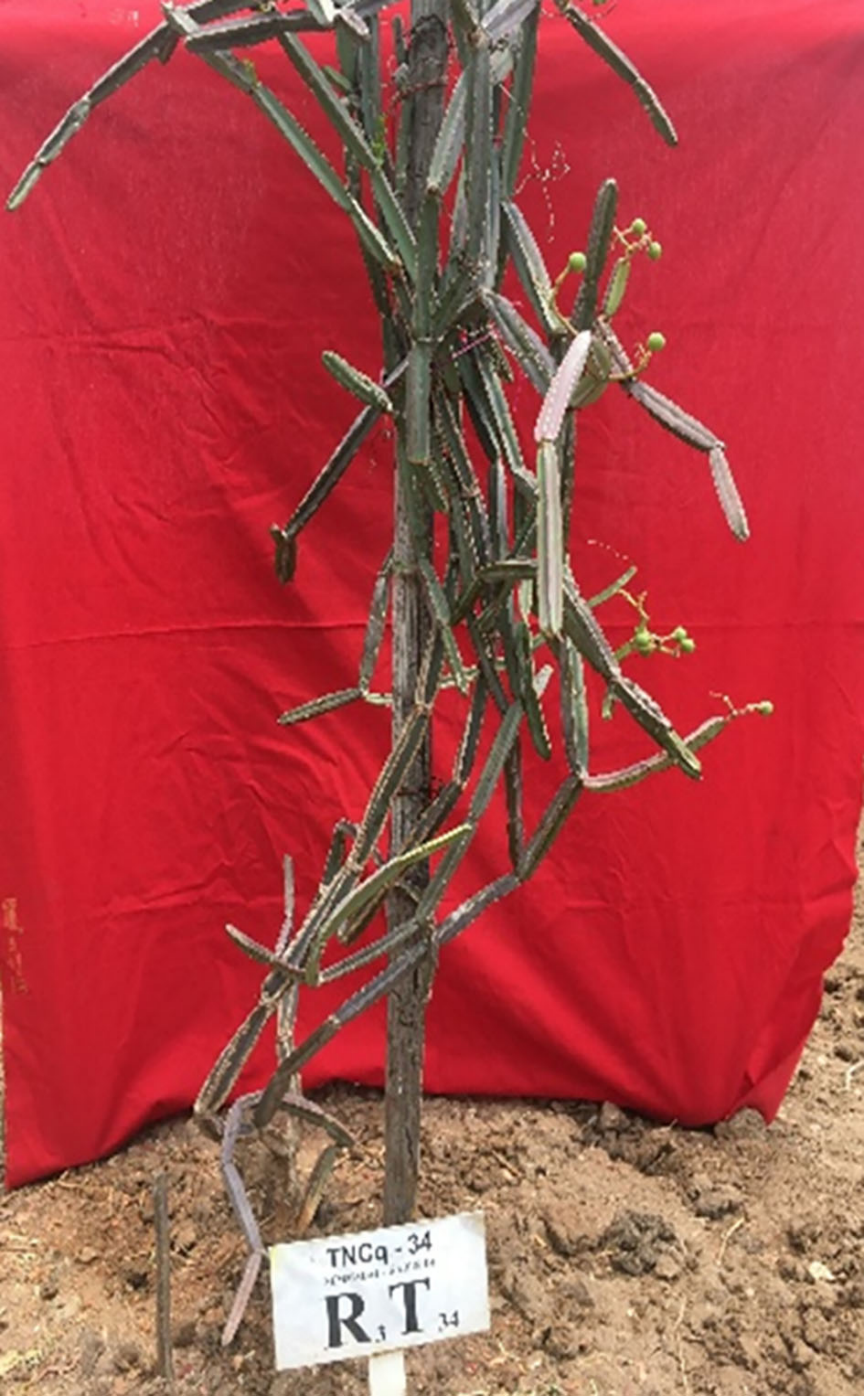
TNCq34 — Berry bearing ecotypes of *C. quadrangularis*. TNCq, Tamil Nadu *Cissus quadrangularis* 1 (Vinoth et al., 2021).

The berry is obovoid or globose, roughly 6 mm long, apiculate, red when ripe, and single-seeded. **Figure 9** shows the flowering ecotype, and **Figure 10** shows the berry-bearing ecotypes.

Morphologically superior ecotypes of *C. quadrangularis* (TNCq 9, TNCq 23, TNCq 28, TNCq 32, TNCq 34) have a high stem yield, plant height, increased petiole length, root weight, root girth, and root length. **Table 3** presents the morphologically superior ecotypes of *C. quadrangularis* (Vinoth et al., 2021).

**Table 3:** Morphological superior ecotypes of *C. quadrangularis* (Vinoth et al., 2021).

Morphologically superior ecotypes	Ecotypes number	Sources
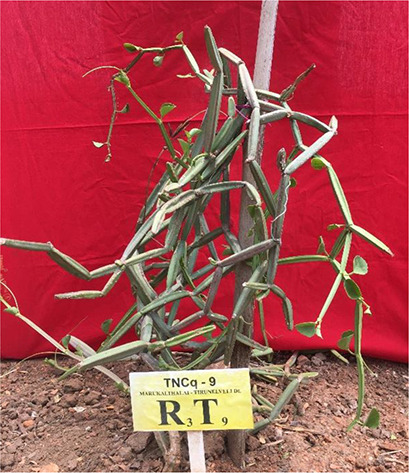	TNCq 9	Marugattuthalai, Tirunelveli
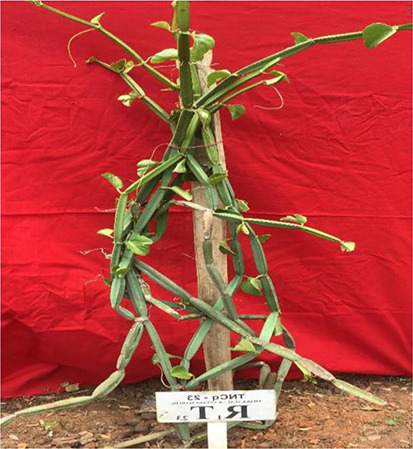	TNCq 23	Thakkalai, Kanyakumari
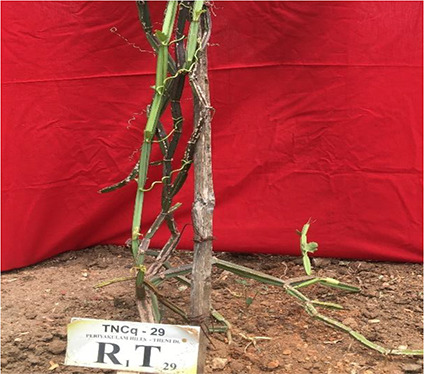	TNCq 29	Periyakulam hills, Theni
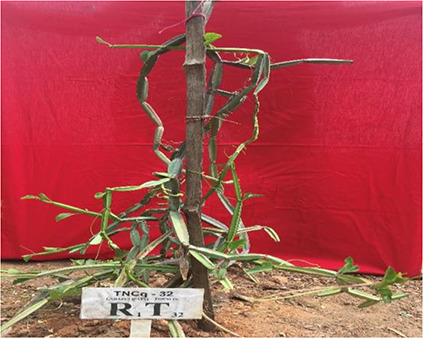	TNCq 32	Endapulippatti, Theni
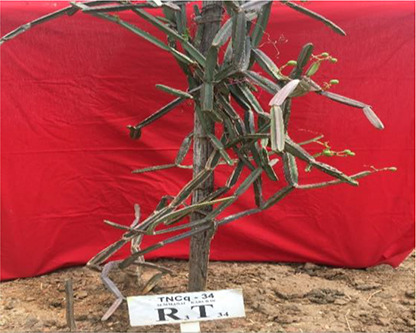	TNCq 34	Semmadi, Karur

## Agronomic and genetic potential

### Vegetative propagation of *C. quadrangularis*

Vegetative propagation can generate true-to-type plants from their parent material rather than seeds. This approach is particularly applicable to medicinal and aromatic plants. *C. quadrangularis* is efficiently reproduced using its mature stem cuttings (Bupesh, 2020).

### Biotic stress in *C. quadrangularis*

The proof for the plant’s response to the biotic stressors is an area that is not properly researched, although a few studies have been conducted, and it has been shown that plants do have interactions with pathogens and pests. *C. quadrangularis* can interact with microbes through antimicrobial activity, and it is thus believed to act against fungi. Lekshmi et al. (2014) have stated that the plant has antioxidant content from the stem extract that is suitable for the plant to face oxidative stress due to biotic factors. A review reported by Bupesh (2020) lists some bioactive compounds of *C. quadrangularis*, including flavonoids and triterpenoids, which may help the plant protect itself from biotic stresses.

### Abiotic stress in *C. quadrangularis*

Crassulacean acid metabolism (CAM) is an effective photosynthetic pathway for conserving water in different plants, including those in the *Cissus* genus (Bone et al., 2015; Nobel, 1991; Sayed, 2001). The facultative crassulacean acid metabolism (CAM) pathway in *C. quadrangularis* could contribute to its excellent tolerance to drought conditions (Ting et al., 1983). *C. quadrangularis* expresses crassulacean acid metabolism (CAM) and C3-like photosynthesis pathway in the stem and leaves, respectively. The plant also employs these two methods to use the low amount of water during the drought times. The CAM pathway can be seen as the most water-efficient pathway *in C. quadrangularis* since it causes the least water turnover (Ting et al., 1983). The plant also acts against the water stress by retaining highly dynamic structures such as polysomes, dictyosomes, and the nucleolus with reduced relative water content as low as 52%–58% (Santo et al., 1980).

## Character association of *C. quadrangularis* through morphological traits

The necessity of preserving the heterogeneity of hereditary factors as a reserve is considered the most crucial step in deciding between crop improvement and coping with the resistance of pathogens in a given population (Idahosa et al., 2010). Genetic variation is the deviation present in alleles of genes or variation in RNA/DNA sequences of different genotypes of the same species, producing distinct phenotypes in a mixed population. Phenotypic variability is partitioned into two components, viz., genetic and environmental influences, which determine the nature and quantum of variability existing among the various economic traits. Detection of diverse lines in an existing population is helpful for the correction of error factors in commercial varieties (Bhanu, 2017). In terms of the association of the character of *C. quadrangularis*, it has been observed that the wild forms are relevant to genetic variability, and ecotypes were found to be significantly diverse for all 17 morphological traits. There were also high estimates of GCV (>35%) available. There was a strong correlation between high heritability estimates (>90%) and high genetic advance as a percent of mean values (>70%) (Vinoth et al., 2021). Likewise, research showed that the heritability estimates of nine morphological characters ranged from 56.37 to 99.27, and genetic advance as a percent of the mean for different morphological traits ranged between 22.10 and 72.90 (Anushma et al., 2021).

## Genetic diversity of the *C. quadrangularis* through molecular traits

Study objectives include morphological, isozyme, protein, and DNA marker assessments to reveal the genetic variation in crops. A vast number of polymorphisms have been revealed, and the advantages of using the information available in the DNA sequence to develop a direct relationship of input between biological function and sequence differences have already been proven by looking at identifying polymorphisms. As discussed by H.D. Mignouna et al. (1998), the collective molecular marker sequence dispersed throughout the genome and its association with polymorphic nucleotide sequences make it possible to determine the genetic diversity of intraspecific species (Gostimsky et al, 2005). In the case of the genetic diversity of the *C. quadrangularis* through molecular markers, a significant amount of polymorphism was found (50.54) by using the Random Amplified Polymorphic DNA (RAPD)– Polymerase Chain Reaction (PCR) technique in the five morphologically superior ecotypes (Vinoth et al., 2021). Remarkably, three morphotypes—morphotype I, morphotype II, and morphotype III—have been distinguished based on the anatomy of the stem and leaves. The three morphotypes have been genetically distinguished using RAPD and Inter-Simple Sequence Repeat (ISSR) molecular markers. In contrast to morphovariant II, which had a flattened stem, morphovariants I and III formed the same cluster (Kaur and Malik, 2014).

## Genome assembly of *C. quadrangularis*

The nuclear DNA content of *C. quadrangularis* was determined to be 2C = 1.11 pg by flow cytometry, with radish as the standard. The DNA amount for the *C. quadrangularis* specimen was found to be 2C = 1.410 pg, which is equivalent to 689 Mbp/L (1 pg = 978 Mbps) (Dolezel, 1997). Based on Soltis et al. (2003), it contains a genome of a size from 1.7 to 3.4 times *Cissus javana* and *Cissus rotundifoli*a with a diploid genome (Chu et al., 2018a).

According to Chu et al. (2018a), *C. quadrangularis* species are characterized by a basic chromosome no. of n = 10, 11, or 12. A study on the genome of *C. quadrangularis* indicated that the chromosomes of the diploid are 2n = 24 and 28, and a sample from India was 2n = 45, suggesting it to be a tetraploid (Raghavan, 1958). However, the differences in several chromosomes could be related to species from different geographical locations that are subjected to epigenetic alterations such as DNA methylation and histone modifications that can either silence transposable elements or drive chromosomal evolution in particular geographical zones (Li et al., 2017b). Given the restricted number of samples that have been studied, the chromosome numbers for *C. quadrangularis* are not clear. A wider range of studies should be carried out to cover different locations by designing the model of the *Cissus*-peptide gene flow and inferring that among the tested individuals, a 2n = 48 chromosomal count is the most probable genotype (Karkamkar et al., 2010b; Chu et al., 2018a). However, further molecular evidence must be generated to confirm this hypothesis.

## Phytochemical composition of the *C. quadrangularis*

*C. quadrangularis* has bioactive compounds such as tannins, phenols, and flavonoids. Similar to other medicinal plants, it also possesses bioactive compounds that are responsible for traditional medicinal uses (Bafna et al., 2021a). According to Bupesh (2020), the active metabolites discovered in the plant play a vital role in various pharmacological activities. Murthy et al. (2003) reported that the plant has many saturated fatty acids, e.g., icosanyl, icosanoate, and *C. quadrangularis* is a natural source of beta-carotene and vitamin C.

The use of the LC-MS technique in the identification of a variety of compounds and terpenoids, including flavonoids, and phytosterols in *C. quadrangularis* has been well documented. It is especially useful for determining substances that include campesterol, β-sitosterol, and stigmasterol in different extracts (Kaur et al., 2022; Aarthi et al., 2024a). *C. quadrangularis* is rich in phytochemicals, and these have been traditionally used to treat diseases and alleviate symptoms of certain diseases (Dhanasekaran, 2020). Flavonoids, in particular, come in contact with the radicals, relaxing the stressed system and thus preventing the persistence of diseases. Phenolic compounds present in the plant are capable of modulating the pathways that expand various therapeutic effects. Triterpenoids that are in the *C. quadrangularis* are another major compound, as they also act as anti-inflammatory and analgesic properties, which greatly relieve the pain and reduce the inflammation (Kumar et al., 2015). The phytochemical composition of this plant points out to be essential for traditional and contemporary medicinal practices (Tiwari et al., 2018). **Table 4** depicts some of the important phytochemical compounds and their chemical structures of *C. quadrangularis.*

**Table 4:** Phytochemical compounds in *C. quadrangularis*.

Compounds Category	Part of the herb	Compound name	Chemical structure	References
Iridoids	Whole herb	Picroside I	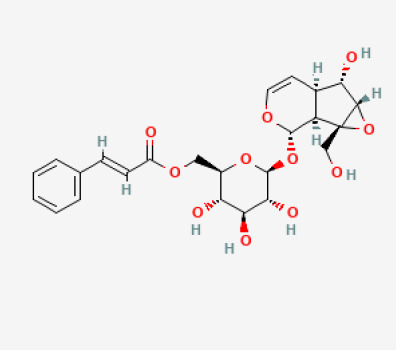	Singh et al. (2007)
Stilbenes	Whole herb	Quadrangularin A	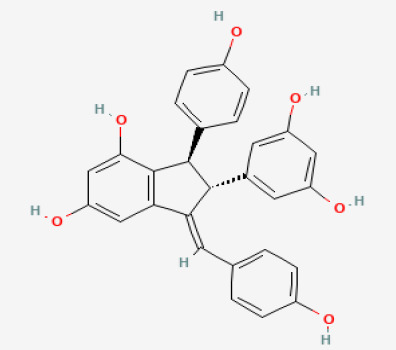	Adesanya et al. (1999); Singh et al. (2007)
	Leaves	Resveratrol	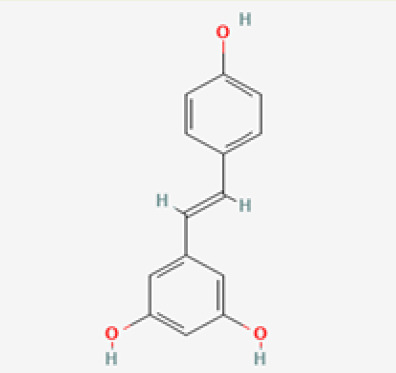	Adesanya et al. (1999); Gupta and Verma (1991)
	Leaves	Pallidol	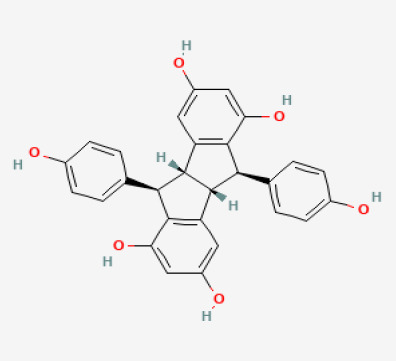	Adesanya et al. (1999); Gupta and Verma (1991); Singh et al (2007)
	Stem	Trans-resveratrol-3-O-beta-glucoside	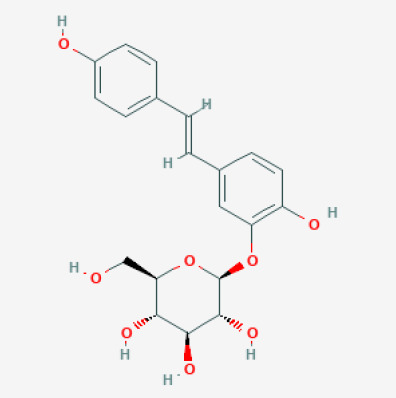	Thakur et al. (2009)
Flavonoids	Whole herb	Quercetin	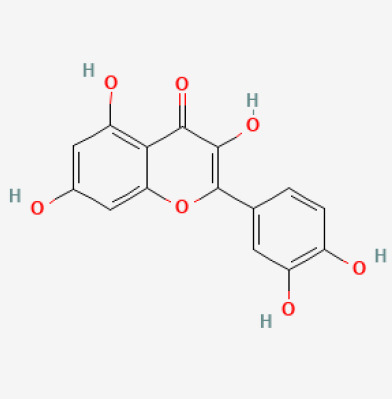	Singh et al. (2007)
	Whole herb	Quercitrin	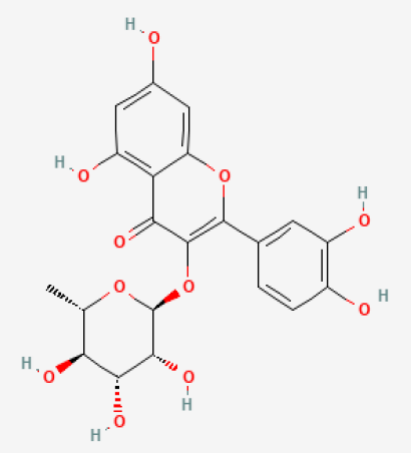	Singh et al. (2007)
	Leaves	Daidzein	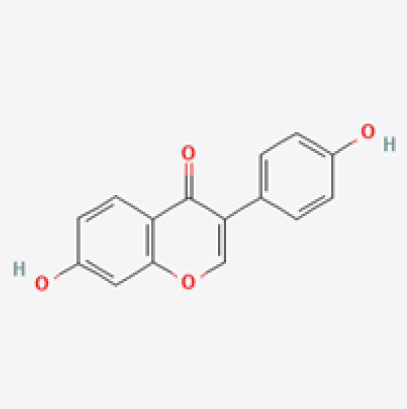	Mukherjee et al. (2016)
Alkaloids	Stem	Kaempferol	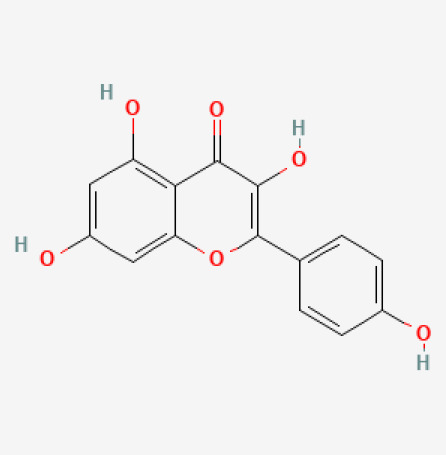	Gupta and Sharma (2008)
	Leaves	Quinine	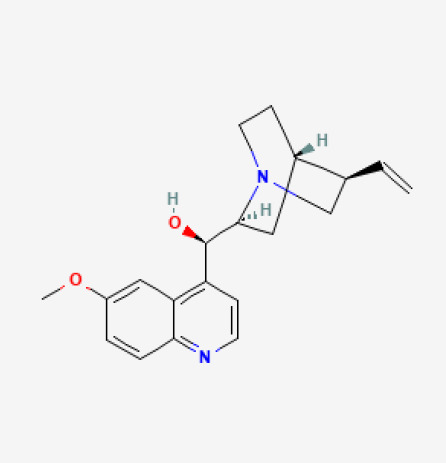	Tiwari et al. (2018)
	Whole herb	Caffeine	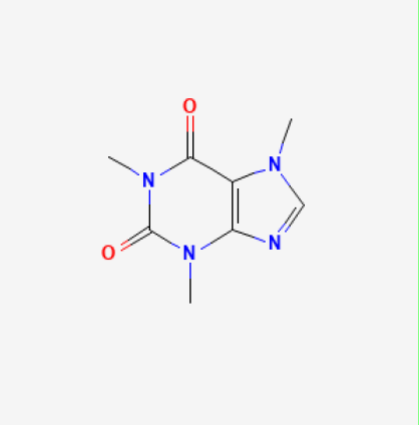	Tiwari et al. (2018)
Steroids	Stem	Beta-sitosterol	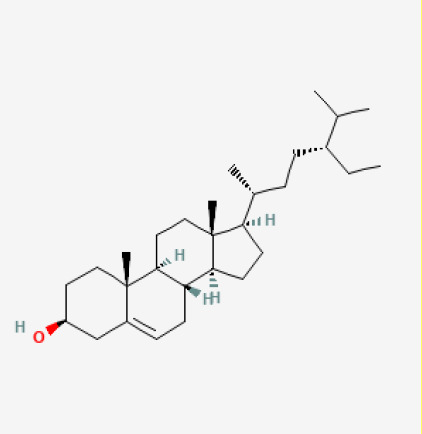	Jainu and Devi (2005); Mishra et al. (2010); Shah et al. (2010); Singh et al. (2007)
Carotenoids	Stem	β carotene	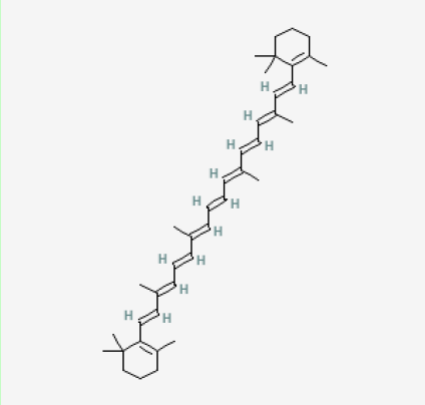	Jainu and Devi (2005)
Vitamins	Stem	Vitamin C	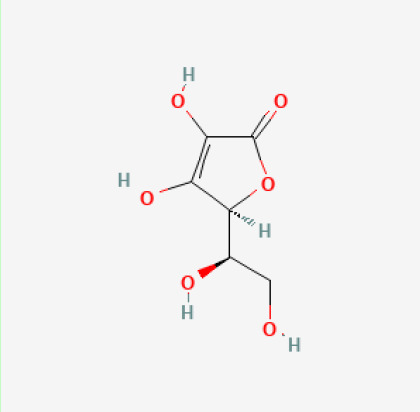	Jainu and Devi (2005)
Saponins	Stem	Saponins	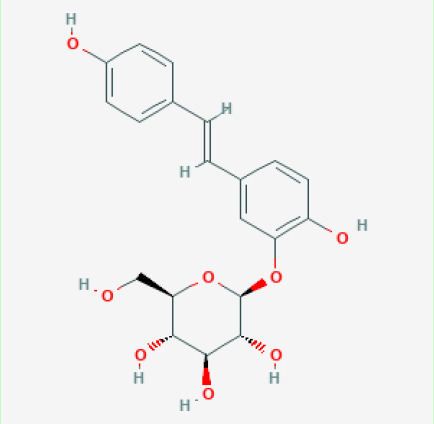	Johns et al. (1999); Teware et al. (2015)
Glycosides	Stem	Cardiac glycosides	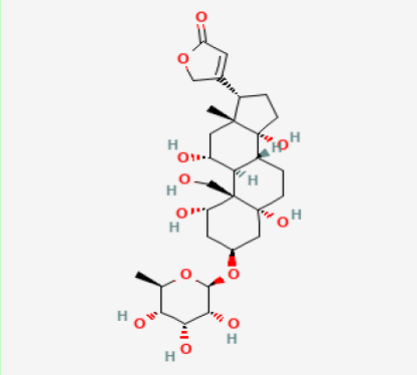	Das et al. (2018); Nagani et al. (2011)

Source: All the chemical structures sourced from https://pubchem.ncbi.nlm.nih.gov.

## *C. quadrangularis* as a bonesetter

**Figure 11:**
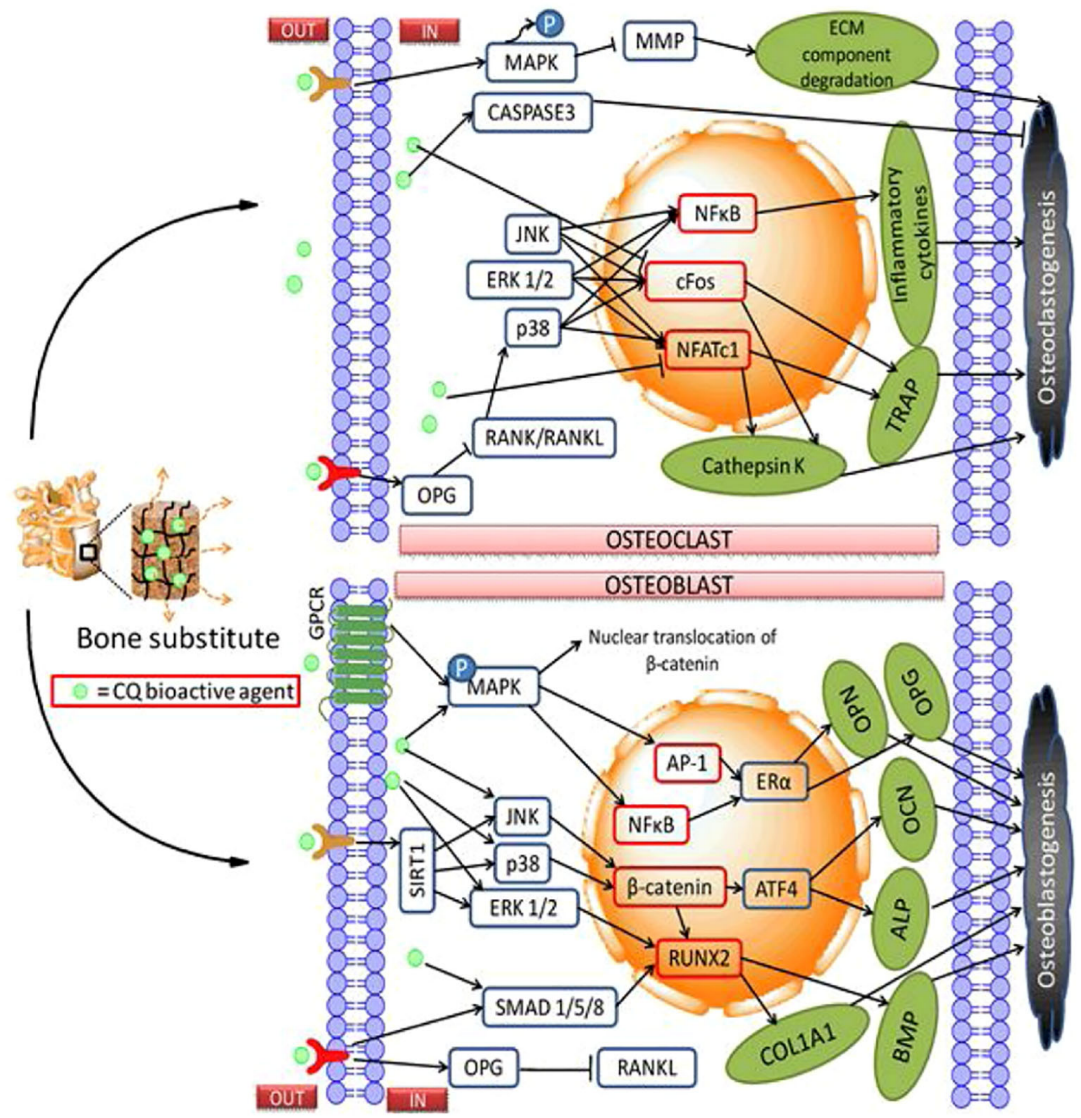
Schematic representation for orthopedic delivery of *C. quadrangularis* bioactive agents. Source: Singh et al. (2020).

Bone fractures are a global public health issue and a significant economic burden, particularly osteoporosis. In the year 2019, globally, there were 178 million new fractures, 455 million prevalent cases (acute or long-term fracture symptoms), and 25·8 million Years Lived with Disability (YLDs) (Hernlund et al., 2013b; Polinder et al., 2016b). Some studies reported that fractures have an important impact on individuals, families, societies, and healthcare systems, resulting in loss of productivity, disability, poor quality of life, loss of health, and substantial costs to healthcare systems (Pike et al., 2010b; Borgstrom et al, 2020a; Tatangelo et al., 2019). *C. quadrangularis* assists with decalcification in the first phase of the healing process, where the little callus is formed. This raises calcium levels sufficiently to unite the two parts of bone, which assists in the remodeling of the bone (Pg et al., 2022). During bone treatment by *C. quadrangularis*, a faster process of calcification was reported, as well as remodification, accounting for rapid healing in animals. Additionally, *C. quadrangularis* can significantly increase the tensile strength of fractured bones to about 90% of normal strength in six weeks (Mishra et al., 2010). *C. quadrangularis* is essential for improving collagen, mucopolysaccharides, calcium, and phosphorus. Mucopolysaccharides are indeed crucial for the healing process of bone. Consequently, during the initial stages of bone remodeling, there is a greater increase in deposition of these substances, which increases the rapid healing rate (Kumar et al., 2018). **Figure 11** is a schematic representation of the orthopedic delivery of *C. quadrangularis* bioactive agents (Singh et al., 2020).

## Anti-osteoporotic activity

*C. quadrangularis* has anti-osteoprotective activity by blocking RANKL-induced osteoclastogenesis, increasing the anti-osteoclastogenic cytokines (IFN-γ, IL-4, IL-10), and decreasing osteoclastogenic cytokines (IL-6, IL-17, TNF-α) and thus improving bone health in estrogen-deficient conditions (Bafna et al., 2021a). *C. quadrangularis* has anti-osteoprotective activity by inhibiting IL-1β-induced cell toxicity, increasing surviving expression, and promoting bone and cartilage regeneration through the p38 MAPK pathway, thus increasing alkaline phosphatase and tissue formation in osteotomized rats (Kanwar et al., 2015). The hexane extract of *C. quadrangularis* showed anti-osteoporotic activity in ovariectomized mice by increasing bone mineral density, restoring trabecular bone, and decreasing serum osteocalcin and TRAP5 b levels without toxicity (Pathomwichaiwat et al., 2012).

In the ovariectomized rat model of osteoporosis, the anti-osteoporotic activity of ethanol extract was evaluated at two different doses of 500 and 750 mg/kg. Raloxifene (5.4 mg/kg) and *C. quadrangularis* ethanol extract (500 and 750 mg/kg) were given orally to the test groups. Ethanol extract showed anti-osteoporotic effect based on biochemical and histopathological parameters (Shirwaikar et al., 2003b). A study by None Keshamma (2022) reported that *C. quadrangularis* has anti-osteoporotic activity by protecting bone microarchitecture, reducing inflammation, and modulating bone morphogenetic protein and Wingless-related Integration Site (WNT) signaling pathway, thus decreasing osteoclastic activity and promoting bone formation, and thus a potential therapeutic for osteoporosis.

## Gastroprotective and antimicrobial effects

Phytochemicals that include flavonoids and triterpenoids from *C. quadrangularis* are responsible for effectively killing bacteria of different types, thus making it a powerful medicine for stomach and bowel problems. The traditional Ayurvedic system supports the properties of the plant, which is used in the prevention of gastrointestinal disorders (Camil et al., 2020). The research study conducted by Nair et al. (2024) explains the idea that *C. quadrangularis*, as a result of the potential of antibacterial and antifungal properties, can destroy *Porphyromonas gingivalis*, which is the key pathogen causing periodontal disease. The ethanol and water extracts of *C. quadrangularis* have also shown the ability to kill the bacteria, although ethanolic extracts showed higher activity against *P. gingivalis.* In either of the cases, a minimal growth inhibitory concentration of 500 µg/mL was identified, suggesting the fact that bacteria would no longer be able to grow at this concentration. *C. quadrangularis* has been shown to significantly reduce aspirin-induced gastric lesions as it may help to shield the stomach lining from injury due to non-steroidal anti-inflammatory drugs (NSAIDs). Sadiya (2020b) have shown that *C. quadrangularis* inhibits levels of pro-inflammatory markers such as TNF-alpha and myeloperoxidase. Reducing these markers is critical for preventing the inflammation of the stomach, which can result in ulcers and further gastrointestinal issues. **Table 5** lists some of the antimicrobial and antifungal activities of *C. quadrangularis.*

**Table 5:** Antimicrobial and Antifungal activity of *C. quadrangularis*.

Antimicrobial activity of *C. quadrangularis* (George and Kashikar, 2006)			
	MIC values in mg/mL		
	Ethyl acetate	Methanol	Acetone
*Bacillus subtilis*	0.93	0.465	
*Pseudomonas aeruginosa*	1.87	3.12	3.125
*Salmonella typhi*	3.75	1.24	6.25
*Staphylococcus aureus*	0.93	0.465	1.56
*Streptococcus pyogenes*	3.75	0.93	
			
Antifungal activity of stem methanol extract of *C. quadrangularis* (Payani et al., 2023)			
	Zone of inhibition (mm)		
	Concentration (µg/mL)		
	1000	750	500
*Candida albicans*	14	12	11
*Penicillium crysogenum*	7	7	7
*Trichoderma viride*	13	11	9

## Antifungal and antibacterial activity

The 90% methanol extract, along with a dichloromethane extract from the stems, has shown that they can be effective against bacteria such as *Staphylococcus aureus, Escherichia coli*, and *Pseudomonas aeruginosa* and also cause mutations in the Salmonella microsomes. The stem and root extracts have been shown to have significant antibacterial properties as well. The ethanolic extract from the above-mentioned plant parts has also been proven to counter the parasite *Entamoeba histolytica*. The stem alcoholic extract also showed its effectiveness against *E. coli* (Chenniappan et al., 2020a). *In vitro* methods were applied to the evaluation of the antimycotic activity of the water, butanol, and acetone extracts obtained from *C. quadrangularis*. During the study, it was established that the N-butanol extract was the most effective on *Geotricum candidum* (19.8 ± 0.1) inhibitor. Water extract was able to deal with *Penicillium* species (9.1 ± 0.2), while the acetone extract prevented *Aspergillus flavus* from growing. Ethyl acetate extract from endo-fungus ACQR8 has the most varied antimicrobial effects (Singh et al., 2021).

## Future insights

India has a rich diversity of *C. quadrangularis* ecotypes, but there are limited morphological and molecular studies available for *C. quadrangularis* that show none of the varieties released up to the present. Further study is required for linking the molecular markers to morphological traits with pharmacologically active ecotypes. Investigation on phenotypic plasticity of the plant will help in figuring out the influence of environmental factors on its morphological character and the secondary metabolite accumulation. In the case of molecular aspects of the plant, it is necessary to analyze the whole genome sequencing and transcriptome profiling for the genes and proteins involved in the accumulation of the principal phytochemical compound of the plant.

## Conclusion

In conclusion, the present review covered the comprehensive information on the morphological, molecular, and pharmacological aspects of the plant. The genetic diversity studies using morphological traits unveiled the existence of a high degree of variability among the *C. quadrangularis* ecotypes, which can be well utilized for the formulation of suitable strategies for improving economic traits of the plant. CAM mechanism is a major photosynthetic pathway that enables the plant to survive in stressed conditions to adopt wide range of climatic conditions. It is also a potentially underutilized medicinal plant. It has promising phytochemical profiling that is used in various pharmacological studies, including the anti-osteoporotic activity and antimicrobial activity. Although the plant has a huge, diverse population with significant bioactive compounds, there are fewer studies on the selection of the superior plants from the wild form to the cultivated one. Henceforth, further studies are needed concerning the morphological and molecular levels for conserving the plant genetic resources and crop improvement program.
